# Reviewed article: Santos DS, Viana PVS, Basta PC. Observational study of the coverage rate and survival analysis of COVID-19 vaccination among Indigenous people in Roraima, 2021-2023. Epidemiol Serv Saude. 2025:34;e20240762

**DOI:** 10.1590/S2237-96222025v34e20240762.b

**Published:** 2025-10-24

**Authors:** Matheus Santos Melo

**Affiliations:** 1 Ministério da Saúde, Brasília, DF, Brazil

## Peer review B

## Questionnaire

Does the manuscript contain new and significant information to justify publication? Yes

Does the Abstract (Summary) clearly and accurately describe the content of the article? No

Is the problem significant and concisely stated? Yes

Are the methods described comprehensively? Yes

Are the interpretations and conclusions justified by the results? Yes

Is adequate reference made to other work in the field? Yes

## Manuscript Structure

Length of article is: Adequate

Number of tables is: Adequate

Number of figures is: Adequate

Please state any conflict(s) of interest that you have in relation to the review of this paper. None

Interest: Good

Quality: Average

Originality: Good

Overall: Average

Recommendation: Major Revision

## Comments

O artigo aborda um tema relevante para uma população vulnerabilizada.

Existem pontos que poderiam ser aperfeiçoados:

1) Título

O título possui termos dispensáveis e não informa tão bem o propósito do artigo. Sugiro algo como “Cobertura vacinal contra a COVID-19 em indígenas de um estado da região Amazônica brasileira: uma análise de sobrevivência de 2021 a 2023”

2) Resumo

Sugiro que deixem claro qual estudo epidemiológico que o artigo se enquadra. Observacional de que tipo (transversal, coorte....)?

3) Objetivos

Não é necessário “novo coranavírus” poderiam escrever diretamente “contra a COVID-19”. Outro ponto, é que da forma como está escrito, não deixa claro para o leitor para o quê a análise de sobrevivência foi aplicada. É necessário ler os resultados para entender isso. Sugiro que deixe claro.

4) Introdução

A relevância de se comparar diferentes grupos indígenas não está clara.

5) Métodos

Sugiro esclarecer qual tipo de estudo epidemiológico se enquadra. Seria relevante realizar a análise de sobrevivência para toda a população e não apenas estratificada. Também seria interessante realizar análise de COX para compreender se não existem outras características que explicam as diferenças encontradas entre as sobrevivências.

6) Resultados e discussão

As tabelas estão incompletas, não apresentam n, % em todas as colunas que se fazem necessários, não apresentam legenda. O título da figura deveria ser posto abaixo da mesma.

Sugiro que a limitação seja revista, principalmente, ao considerar as técnicas estatísticas utilizadas. Já que a técnica não é suficientemente robusta (segundo o que parecer ser sugerido pelos autores) para o que os autores se propõe, não seria interessante utilizar outras? Além disso, “altamente significativos” não é um termo apropriado. Isso pode ser considerada uma falácia sobre a interpretação do p-value. Poderia ser apenas “significativo”.

Os autores comparam o estudo considerando que a literatura “corrobora” com os resultados encontrados na pesquisa, no entanto, deveria ser feito o oposto. Ou seja, o artigo que corrobora ou refuta o que a literature vem descrevendo, favor rever.

Senti falta na discussão de discutir os resultados do trabalho, principalmente o relacionada diferença encontrada na análise de sobrevivência. O por quê dessa diferença e em que essa informação pode agregar a literatura e orientar as políticas públicas.

Qual a relevância de se discutir e tentar justificar o tipo de vacina administrada? Isso não estaria muito mais relacionado ao tipo de vacina disponibilizado do que o interesse da população em recebê-la? Qual o significado das diferenças entre as zonas de residência?

Qual a repercussão dos achados para construção de políticas públicas? Como eles deveriam ser utilizados?

O que a literatura ainda precisa avançar sobre a temática?

